# Maintaining well-being during COVID-19: a follow-up study of community dwelling older people in New Zealand

**DOI:** 10.1177/17449871251342179

**Published:** 2025-07-02

**Authors:** Susan Waterworth, Deborah Raphael

**Affiliations:** Senior Lecturer, School of Nursing, Faculty of Medical and Health Sciences, The University of Auckland, Auckland, New Zealand; Research Fellow, School of Nursing, Faculty of Medical and Health Sciences, The University of Auckland, Auckland, New Zealand

**Keywords:** COVID-19, older people, qualitative study, well-being

## Abstract

**Background:**

Older people are often viewed as a vulnerable group, particularly during the COVID-19 pandemic, with associated images of passivity and lack of agency. In contrast, we know that older people are resilient and have ways of managing adversity. This study aimed to explore how older people managed their well-being during the COVID-19 pandemic in New Zealand.

**Methods:**

This study applied a unique approach by following up a sample of older people from our previous research exploring their well-being and how they managed their well-being. Semi-structured interviews were conducted building on the participant’s previous interviews and earlier responses.

**Results:**

Fourteen participants participated in the follow-up study. Despite the challenges associated with lockdown, participants were creative in adapting to their situation, maintaining their positivity, connecting with others using social media, and continuing to engage in activities they valued.

**Conclusions:**

Understanding the approaches and factors that influenced older people’s well-being can be a guide for nurses working with older people, providing health communication and adapting approaches to meet their needs. The need for access to technology and being able to use the technology to enhance well-being practices are important factors. As disparities can arise, in for example access and ability to use technology, individualised assessments by nurses are required.

## Introduction

During the COVID-19 pandemic, older people were, and continue to be, identified as a high risk and vulnerable population (Brooke and Clark, 2020; Yang et al., 2021). Most of the literature on COVID-19 and older people has focused on their levels of fear and anxiety; and concerns about the impact on older people’s mental and physical health, particularly amongst those who are isolated (Armitage and Nellums, 2020). Internationally, mortality rates associated with COVID-19 have been high, particularly for older people (Yanez et al., 2020). Regular reporting of international COVID-19 deaths may have added to the fear and anxiety, and the research focus on measuring negative outcomes for older people (Pinedo et al., 2021).

A review of news media during COVID-19 in New Zealand found older people were represented as a nameless, homogenous ‘other’ group with under-representation of their agency and ability to navigate the pandemic (Morgan et al., 2021). Derrer-Merk et al. (2022) argued that the protective measures introduced during the pandemic created a new form of ageism. Another review found mixed evidence on whether age was actually a risk factor for well-being during the COVID-19 pandemic (Okabe-Miyamoto and Lyubormirskly, 2021).

Adherence to guidelines became not only an expectation but also a requirement in legislation, despite the recognition that these could mean people experienced losses, for example, in resources (Yagil and Cohen, 2022). In New Zealand, on 21st March 2020, people over the age of 70 years were advised by the Prime Minister Jacinda Ardern to remain at home, and the country introduced its 4-level alert system (Ministry of Health, 2020). Level 4, the highest level requiring everyone to stay at home and self-isolate, except essential workers, occurred on the 26th March 2020. The rules of alert Level 4 included keeping your household bubble as small as possible and only making physical contact with those in your bubble (Table 1).

**Table 1.** Alert Level 4 rules.

We all need to stay in our household bubble.
You cannot invite friends or family to your home.
If you want to talk to a friend, call or video chat with them.
You can exercise in your local area on your own, or with people in your household bubble.
You can drop off groceries to others, but drop the groceries at the door. Always keep a 2 m distance.
You can drive to a place to exercise – for example, to a park – but you should stay close to where you live. Do not travel too far, that is no further than 5 km.

Internationally, during this time, concerns were raised about the term ‘social distancing’ and the risk to the health and well-being of social isolation for older people. Following recommendations, the term physical distancing rather than social distancing was introduced by the World Health Organization to emphasise that it was distance rather than actual contact with others that incurred risk, and the mitigate the associated risks to mental health of social isolation (Wasserman et al., 2020).

The aim of this paper is to present how older people were managing their well-being during the early phase of the pandemic. Well-being in this paper is based on Seligman’s theory of well-being, reflecting both the eudaimonic and hedonic dimensions of well-being. The hedonic approach focuses on happiness and defines well-being in terms of pleasure attainment and pain avoidance; and the eudaimonic approach, focuses on meaning and self-realisation and defines well-being in terms of the degree to which a person is fully functioning (Hone et al., 2014).

## Methods

This qualitative descriptive study involved following up a sub-sample of older people from our previous community well-being study (Waterworth et al., 2019). The follow-up study was conducted once face-to-face interviews could be completed during 2021. The COREQ checklist for reporting qualitative research (Tong et al., 2007) was followed (Supplemental File 1).

## Design

The design was informed by our previous study exploring how older people managed their well-being and informed by Seligman’s PERMA framework for well-being (Kern 2022). Seligman proposed the building blocks of well-being as consisting of five elements: positive emotion, engagement, relationships, meaning, accomplishment, and subsequently ‘health’ was added (Kern 2022). A person can take positive action within each element, and each element can be interrelated. For example, being engaged in a physical activity group may involve maintaining relationships with other people and create meaning in the person’s life. Within each element, different approaches or positive interventions may be used, reflecting what matters to the individual. Ethical approval was obtained from The University of Auckland Ethics Committee (024624).

## Participants

We identified people over the age of 75 from the original study who had shown interest in further follow-up studies on their well-being. Our initial list of 20 people who had expressed interest was reduced to 14 as some of our previous participants had died or were too unwell to participate in further research. Information was either posted or emailed to potential participants and followed up with a telephone call either by (SW) or (DR) giving participants the opportunity to ask questions. All participants signed the consent form and either posted or returned the form electronically. Alert levels in relation to pandemic measures changed during the study period, and some interviews were conducted face to face in the person’s home (*n* = 9), and other interviews conducted by telephone (*n* = 5). All interviews were audio-recorded with permission from each participant, and the recording stopped if the participant did not want specific material recorded. The 14 participants who took part in the follow-up study provided a diverse sample of older people. All the participants lived in the city of Auckland, eight were female and six were male. Age ranged between 76 and 91, with nine participants over the age of 80 years. All participants had at least one long-term condition, ranging from 1 to 4. Six participants were married, three divorced, three widows and two widowers. Eight participants were living alone.

We knew the sample of 14 participants could provide information to meet the aim of the study. Malterud et al. (2016) identified five items across a number of dimensions that impact the information power of the sample: the aim of the study, sample specificity, use of established theory, quality of dialogue and analysis strategy. *Study aim* – Our study aim was narrow to specifically explore with the participants how they had managed their well-being during the early phase of the COVID pandemic. *Sample specificity* – Our choice of participants was based on our previous study on how older people maintained their well-being. Recruiting from this sample of participants meant we had to take a pragmatic approach and interview those who had previously agreed to be contacted about their well-being in the future. We had data on how they managed to achieve their well-being. *Use of established theory* – Seligman’s PERMA framework for well-being informed our approach and had been used in our previous study (Kern, 2022). In Seligman’s theory there are five building blocks that enable ‘flourishing’ – positive emotion, engagement, relationships, meaning, and accomplishment, hence the acronym ‘PERMA’. *Quality of dialogue* – relationships were already established with the participants and knowing there could be clear communication between the participants and the two researchers. *Analysis strategy* – reflexive thematic analysis (Braun and Clark, 2019) informed our analysis. We also provided information on how we involved the participants in providing feedback on our analysis.

## Data collection

The semi-structured interview guide was based on reading the participant’s previous interviews and reflecting with them on their earlier responses, *identifying any changes arising* during the lockdown periods. The researchers were experienced interviewers and interviewed the same participants, having already built relationships with them. This provided the context for the interviews and was important as we were using information that the participants had previously shared with us. In effect, acknowledging their voices and previous contributions. We were now exploring if there had been any changes in their *positive* well-being practices, importantly what was still working or not working well and the barriers or risks to maintaining their well-being during the pandemic.

We asked about any changes in health since their last interview and participants completed the four-item Patient Health Questionnaire (PHQ4), construct validity and mental health (0.80) (Stanhope, 2016). The questionnaire was only used as a guide to generate conversations about the participant’s emotional state, for example, potential feelings of anxiety, worry, depression or a lack of interest in usual activities.

## Data analysis

**Figure 1.**
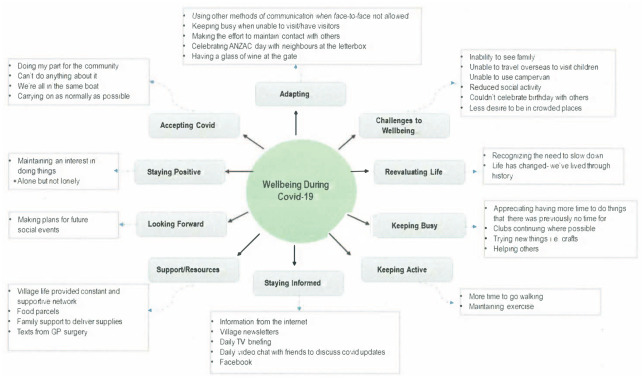
Mind map.

A process of reflexive thematic analysis (Braun and Clark, 2019) was completed. Each researcher reviewed the transcripts, developed codes and formulated a mind map to represent the ideas, concepts and themes within the data (Wheedon and Ahlberg, 2014). Coming together to compare and revise mind maps led to the creation of an overarching mind map (Figure 1). Using this approach, we were able to determine the links and overlaps between the five aspects of PERMA, *identifying the approaches taken to maintain well-being and the challenges participants were encountering during the COVID-19 pandemic.* Several participants provided feedback on the overall mind map and required no additions or alterations to the map. All participants received a final copy of the results in the form of a well-being booklet that they could share as a resource with other people. The booklet illustrates what we had learned from them about maintaining well-being during the COVID-19 pandemic.

## Results

The results are presented for each of the elements of the PERMA model of well-being with evidence of how they connect or interrelate. Critically, we show how feeling safe and managing uncertainty weave their way through the participants’ well-being. In presenting quotes from our participants, the following information is provided in brackets: the first letter refers to gender, the second letter to marital status, and the final number is the age of the participant at the time of the interview.

### Positive emotion

Despite the lockdown levels and having to remain at home, particularly during Level 4, all participants managed to remain positive with specific ways of managing their emotions. This was influenced by all participants’ perceptions that they were safe in New Zealand. The feeling of safety reduced some of the uncertainty, particularly as alert levels changed.

*‘We are lucky here in NZ to be able to contain it the way we do. I was talking to my niece in … and they just seem to have been in lockdown forever and can’t see a future without it.’* (F.W.88)

It was evident that feeling grateful for the positive aspects of their life was a way of managing in general, not only during COVID-19. As our following participant, a widow who lived alone shares:

*‘One of the things I do, I’m very mindful of is, that I have so many blessings. I never cease to be thankful for that. If I should feel down, which honestly I can’t tell you when I last felt down, I would go for a walk or I would put on some music. Or I would lose myself in, in reading something positive and worthwhile.’* (F.W.85)

Whilst keeping up to date with what was going on, all participants were cautious about listening to or watching media that could be providing incorrect or dramatic information. Participants acknowledged that some information, or indeed, misinformation, could generate negative feelings and emotions, engendering uncertainty and the risk of feeling overwhelmed and helpless. The provision of information by people they viewed they could trust was valued.

*‘I found that those TV programmes at one o’clock with Jacinda (Prime Minister of New Zealand and Dr Bloomfield (Director General of Health). I thought they were very, very helpful. I watched them every day and then straight after that one, two, three, four friends and myself we’d keep in touch every single day and every single night and we’d video chat about what we’d heard.’* (F.D.83)

Maintaining positivity was important, and participants had strategies to manage their feelings, they had time to process negative emotions as a normal part of their life. Trying not to be overwhelmed with negativity was important to our participants.

*‘I couldn’t go and see my friends. I would say that there were times, the odd time when I felt lonely. But then I’d get busy with something in the garden, go into my little workshop and come right, so box on.’* (M.M.88)

Concerns were expressed about safety once the lockdown levels changed, as people could go out more. As one participant reflects on the first time she went out on her own:

*‘I went out for the first time I just couldn’t believe how I felt, like I was an alien walking the streets and I just could not wait to get back home to my bubble.’* (F.W.80).

Another participant spoke about how she took extra precautions when going to the supermarket on her own:

*‘When I go to the supermarket, people might think I’m nuts, but I wash the handle of the trolley and my hands. And I’ll get a plastic bag, put a plastic bag over the top and I’ll wheel it with a plastic bag, so that it’s only my hands on the plastic bag. I’ve got hand sanitiser, till it comes out of my ears in my handbag.’* (F.W. 79)

### Engagement

Engagement reflects being absorbed, interested, and involved. Going out of the home during Level 4 was restricted, and older people were advised not to leave home unless essential. Despite these limitations, participants attempted to manage this period by keeping active around their homes and being creative about what could be achieved. For instance, a participant unable to leave her home created the following variety in her activities:

*‘I had various stations to go to. I had my knitting, or I had sewing up, or I had a jigsaw, or I had a book, television or music, or I had a sleep. So, I broke up my day into bits so that it wasn’t such a long day.’* (F.W.82)

Despite the advice to stay at home, the importance of maintaining physical activity was valued, so walking around the neighbourhood for those who could walk was achieved, ensuring safety measures were in place:

*‘I still used to go for a walk reasonably frequently.; I used to go about eight o’clock, 8.30 in the morning and make sure no one was around. I didn’t go near anyone, and they didn’t come near us. We had to watch a little bit to make sure we were wearing masks around the place.’* (M.W.91)

Having options to vary activities such as shopping online and using technology to find information, and keep in touch with family and friends, was essential, for example:

*‘I did read quite a bit. I belong to the Libby, which is the library on the computer. If I couldn’t be bothered looking at the page because my eyes weren’t that good I’d get an audio one. I’d sit on my bed and listen to, or in a chair, and listen to an audio book. So I went through quite a few books.’* (F.W.80)

From our previous interviews with participants, we knew they were contributing to their communities, such as volunteering; or taking on roles in interest or social groups. However, lockdown had made people aware of how much time they had committed to their various social and volunteer roles, and they felt that it was important not to over-commit in the future. Thinking about their life in the post-COVID future included less commitments and wanting more time for themselves and the opportunity to do other things.

*‘The best thing for me that came out of COVID was I realised how very tired I was. And I made the decision to stop the four voluntary jobs I was doing.’* (F.D.85)

### Relationships

Relationships reflect being socially connected and feeling supported and cared about. Bringing a sense of community and connection was achieved in a number of ways and enabled the sharing of challenges that other people were experiencing:

*‘ANZAC^1^ Day was wonderful. On the morning of the dawn service, it was broadcast over the radio. One of the neighbours, a young man about 25, brought the radio out to the middle of the street. We had people out at the gate participating in the service. It worked really well and was good for the street.’* (M.M.79)

Our participants were all able to maintain connections via social media, whether that was via telephone, IPAD/tablet or computer. Whilst the ability to use platforms such as Zoom was limited for a married couple, they still valued being able to connect:

*‘We were not able to go to Church but our Minister sent out a sermon each Sunday and we were able to join in services online.’* (F.M.75)

Participants valued support from certain organisations, for example, the Parkinson’s Society, Age Concern. This involved check-up phone calls to determine how they were doing and whether they needed any support. Missing the opportunity for actual face-to-face contact was accepted, for example, reading groups and knitting groups. However, the importance of physical contact, for example, hugs, was something all our participants missed, particularly from family and friends. The importance of still receiving some home support services, such as assistance with personal care was moderated with accepting support workers wearing protective equipment.

### Meaning reflects a sense of direction and purpose

A sense of purpose for our participants reflected ‘doing their bit’ for the country, adhering to health guidelines in maintaining their own safety, but not putting other people at risk:

*‘I think the first time (lLockdown) everybody felt that you were doing your bit and just wanted to do everything that you were told to do.’* (F.W.89)

Maintaining acts of kindness and helping other people was something that all our participants had identified in our previous study. This continued to happen engendering some different approaches and their strength of creativity. For example, a participant talked about taking flowers and lemons to a couple in an apartment at her village:

*‘They would put a bucket over the balcony, I would fill it and they would pull up the bucket.’* (F.W.79)

### Accomplishment (or Achievement)

Accomplishment reflects continuing to learn and achieve goals and reflects a person’s explanatory style, for example, being optimistic and hopeful about the future. Participants spoke about trying new things or re-starting previous creative pursuits, such as ‘*starting up painting again*’, and we see the overlap between having a sense of direction and purpose with accomplishment. For example:

*‘We’ve got a lady next door, a widow, and she can’t drive her car very well. So the car sits in the garage, it gets a flat battery. So I go next door, charge up the battery, give her car a run. Those are sort of the goals, to keep up with people in the community and help out where I can.’* (M.M.88)

Having survived during the pandemic was seen as an achievement and part of a major historical moment, as the following female participant shared:

*‘We’ve just, all of us have just lived through history. It has changed, and I’m writing my autobiography; I get on the computer every day and write something.’* (F.D.83)

## Discussion

This in-depth study following up 14 participants showed how participants continued to maintain and promote their well-being, potentially minimising the negative impact of living their lives during the pandemic. Their experiences were framed within strategies that worked for them and a unique finding was the creativity participants demonstrated in continuing with simple positive activities. This enabled a sense of routine and control in their everyday living with continuing or adapting activities that worked for them (Kremers et al., 2022; Smith and Hanni, 2019). For example, if tired of reading, then using the audiobook option. The process of savouring positive experiences (Smith and Hanni, 2019) and being able to regulate their feelings was noted by all our participants. That is not to say that negativity was not experienced, but when experienced, it was most commonly due to the uncertainty associated with fluctuations in the lockdown levels and knowing what to do. One of our participants had been receiving face-to-face counselling support for post-traumatic stress disorder, but the face-to-face intervention was discontinued. He was offered contact via Zoom, but at the time he did not think this would meet his needs and so he declined. Moving mental health support to virtual care has been and will continue to be developed (Flint et al., 2020), but how this approach can meet the needs of the older person still requires further testing due to the risk of exclusion (Gorenko et al., 2021).

Maintaining physical activity is recognised as essential for well-being (Bohn et al., 2021), and as in other studies, remaining active is important for health in general (Brooks et al., 2022). In our study, walking was the main way participants achieved their physical activity, including the ability to be out of their home and in touch with nature. For those people unable to leave their home without support, approaches to maintaining physical activity are required. For example, within New Zealand, a TV programme was developed, Healthy for Life, to support older people over 65 to keep active at home during periods of lockdown (https://www.brainresearch.co.nz/news-events/healthy-for-life-new-tv-programme-helps-older-people-stay-active-at-home).

### Uncertainty

Being able to handle and tolerate uncertainty was and still is central to how all our participants were managing to maintain their well-being. Uncertainty is part of life, and the pandemic continues to raise questions, particularly as further variants emerge and concerns about future pandemics are raised with lessons for future planning. Tolerating uncertainty is associated with improved well-being, whereas intolerance of uncertainty negatively impacts well-being with higher risk of anxiety and depression (Satici et al., 2020). Whilst our participants were experiencing uncertainty, they had ways and means to manage this. Some of the methods they used were ensuring they received information from reliable sources and maintaining connections with people they respected and valued so they could share and discuss information.

Traditional social media such as the TV and telephone were helpful, particularly the daily televised COVID-19 update. All our participants were able to use social media and utilised the latest technology to connect and engage with others, particularly family members and friends. For our participants, there was no digital exclusion and risk of internet-related social exclusion (Seifert et al., 2019) as they were able to connect in different ways to support their well-being. The risk of a well-being divide was therefore reduced as they could access appropriate technology (Araki, 2022). Health professionals should assess the preferences and potential barriers to using technology for older people who may be at risk during the pandemic (Gorenko et al., 2021). For example, only providing one option for connection, such as a Zoom video consultation for a psychological intervention, may leave the older person at risk of not meeting their mental health needs (Flint et al., 2020).

‘Being grateful’ and ‘savouring’ are both recognised as cognitive approaches to enhance well-being (VanderWeele, 2020). Expressing gratitude and savouring connections and support from others and being able to give support to others mattered to our participants. The feeling of being important to others brings a sense of being valued and cared for, reflecting a sense of ‘mattering’ (Flett and Heisel, 2020). Similar to other studies (Kremers et al., 2020), our participants felt it was important that they were ‘doing their bit’ for their country by following the advice from health professionals. For some this included being even more stringent about safety procedures even when lockdown levels changed.

Most of our participants were thinking about their future post-pandemic and imagining what they would be doing or doing differently. For example, having more time to be involved in different activities that might be potentially life enhancing (VanderWeele, 2020).

Maintaining religious attendance is important on several levels, having a sense of purpose and meaning in life, connections with others, and health in general (VanderWeele, 2020). Although our participants were unable to attend face-to-face religious services, the option provided by their church for engagement, connection and sharing faith with others was valued. However organisations providing online services need to check whether people can access their specific programmes. Whilst physical distancing was limited, a unique finding was the importance of physical contact with someone else (e.g. hugs). All our participants could not engage in some of their previous activities that would have enabled physical touch, such as celebrating birthdays, in the traditional way with family physically present. The absence of physical touch has been shown to be especially challenging in cultures where touching someone’s body, for example, hands and faces, is meaningful and engenders feelings of comfort and security (Pinedo et al., 2021). Certainly, within New Zealand, Māori cultural practices associated with well-being such as hongi (pressing of noses) were discontinued (Dawes et al., 2021).

The use of humour, an emotion-focused coping strategy, was identified in our previous study as important for maintaining well-being. Again, it was acknowledged as vital by our current participants. In reviewing all the transcripts, we noted periods of laughter by participants and with the researchers conducting the interviews. Humour is a resilience factor that provides a means of detracting from negative thinking, reducing anxiety and tension in older people (Berk, 2015; Monahan, 2015).

Awareness of well-being strategies and being able to assess well-being, what is working and not working during the pandemic can help health professionals in assessing public health initiatives in pandemic planning and creating resources and options that could be beneficial to older adults. Managing uncertainty by asking the question about how the older person is experiencing uncertainty is a critical question for all health professionals.

### Strengths and limitations

Our sample consisted of people who we already knew had ways of managing their well-being and may not be characteristic of other community dwelling older people. For example, they had access to and used online technology. They were able to leave their homes, even for short walks outside, and they had supportive families. However, it was our intention to explore how living during the pandemic and the limitations that lockdown imposed impacted this groups’ well-being. We knew from our past study, the ways they thought about their well-being and what mattered to them. We had built a relationship with the participants, so they were open to sharing their ongoing thoughts and the challenges of the pandemic and how their experiences could be shared with other older people.

## Conclusion

Despite the constraints and disruptions of living during the pandemic, our participants demonstrated an active approach to maintaining their well-being, they were creative and adaptive in activities knowing what would work for them. Feeling safe and managing the uncertainty of the pandemic weaved throughout PERMA criteria, illustrating how activities or interventions within each of the elements can contribute to reducing uncertainty. We found our participants valued being provided accurate information by a regular televised update fronted by people they respected, trusted, and valued. This is a critical factor for health promotion communication. This understanding is critical to inform policy and healthcare initiatives during the pandemic, particularly if healthcare measures introduced as pandemic planning may have unintended negative consequences.

**Key points for policy, practice and/or research**

- Assessing how older people are managing the uncertainty that can arise during pandemic situations and respond to restrictions on their lives is fundamental to mitigate negative effects on their well-being.
- Nurses are key in determining the value of technology in health care management; and assessing the accessibility, availability and affordability of specific technology with older people.
- Assessing and implementing wasy in which older people can maintain their activity levels or modify them during pandemic related restrictions is essential.

## Supplemental Material

sj-pdf-1-jrn-10.1177_17449871251342179 – Supplemental material for Maintaining well-being during COVID-19: a follow-up study of community dwelling older people in New Zealand Supplemental material, sj-pdf-1-jrn-10.1177_17449871251342179 for Maintaining well-being during COVID-19: a follow-up study of community dwelling older people in New Zealand by Susan Waterworth and Deborah Raphael in Journal of Research in Nursing

## Note

1. Australian New Zealand Army Corps (ANZAC) – a national day of remembrance in Australia, New Zealand, and Tonga that broadly commemorates all Australians and New Zealanders who served and died in all wars, conflicts, and peacekeeping operations.
